# AE-YOLO: Research and Application of the YOLOv11-Based Lightweight Improved Model in Photovoltaic Panel Surface Intelligent Defect Detection

**DOI:** 10.3390/ma18235404

**Published:** 2025-11-30

**Authors:** Bin Zheng, Yunjin Yang

**Affiliations:** School of Intelligent Manufacturing, Panzhihua University, Panzhihua 617000, China; yyj021122@163.com

**Keywords:** Adown, efficient channel attention, feature enhancement, photovoltaic panel, YOLO, ablation experiment

## Abstract

With the rapid development of renewable energy, surface defect detection of photovoltaic panels has become an important link in improving photoelectric conversion efficiency and ensuring safety. However, there are various types of surface defects on photovoltaic panels with complex backgrounds, and traditional detection methods face challenges such as low efficiency and insufficient accuracy. This article proposes a lightweight improved model AE-YOLO (YOLOv11+Adown +ECA) based on YOLOv11, which improves detection performance and efficiency by introducing a lightweight dynamic down-sampling module (Adown) and an Efficient Channel Attention (ECA). The Adown module reduces the complexity of computational and parameters through steps such as average pooling preprocessing, channel dimension segmentation, branch feature processing, and feature fusion. The ECA mechanism enhances the model’s response to defect sensitive feature channels and improves its ability to discriminate low contrast small defects through adaptive average pooling, one-dimensional convolution, and sigmoid activation. The experimental results indicate that the AE-YOLO model performs well on the PVEL-AD dataset. mAP@0.5 reached 90.3%, the parameter count decreased by 18.7%, the computational load decreased by 19%, and the inference speed reached 259.56 FPS. The ablation experiment further validated the complementarity between Adown and ECA modules, providing an innovative solution for real-time and accurate defect detection of photovoltaic panels in industrial scenarios.

## 1. Introduction

With the global energy structure shifting towards clean energy, the installed capacity of photovoltaic power generation has experienced explosive growth in recent years. However, photovoltaic modules are prone to various defects such as cracks, thick lines, and short circuits during manufacturing, transportation, and long-term operation, leading to a significant decrease in photoelectric conversion efficiency and potential safety hazards. Therefore, efficient and accurate detection technology for photovoltaic panel surface defects is of significant engineering importance for ensuring power station operation and maintenance efficiency, extending component lifespan, and reducing the Levelized Cost of Electricity (LCOE).

Traditional photovoltaic defect detection methods mainly depend on manual detection or image processing-based algorithms such as threshold segmentation and morphological operations, but these methods suffer from low detection efficiency, high subjectivity, and difficulty in adapting to complex lighting conditions and multi-scale defect features. In recent years, deep learning-based modal, such as Faster R-CNN, have made significant progress in the defect detection. However, existing methods face two core challenges in the photovoltaic scenario. (1) Large-scale variation in surface defects on photovoltaic panels and complex background textures lead to missed detections. (2) The large number of parameters and high computational cost of existing models, make it difficult to meet the real-time inspection requirements of photovoltaic power stations. The YOLO series algorithms stand out in real-time detection tasks due to their efficient single-stage detection architecture, but the basic YOLO algorithm has issues such as insufficient feature fusion capability and inadequate use of contextual information, which still pose bottlenecks in complex defect classification and positioning accuracy.

Due to the similarity, small scale, and complex background of defect features in electroluminescence (EL) image, traditional detection methods face significant challenges. To address this challenge, researchers have proposed various innovative methods based on deep learning. Chen Aidong [1] proposed the improved Faster R-CNN. By integrating lightweight channel and spatial convolutional attention modules, it efficiently analyzes crack defects in complex scenes, optimizes anchor box design using clustering algorithms, and combines DIoU loss function to improve localization accuracy, resulting in a significant increase of 14.87% in average detection accuracy. Su Binyi [2] designed a Complementary Attention Network (CAN), which adaptively suppresses background noise and highlights defect features by sequentially connecting channel attention sub-network and spatial attention sub-networks. Then, the Region Proposal Attention Network (RPAN) embedded with the CAN was constructed, forming an end-to-end RPAN-CNN framework and achieving more refined defect detection on large-scale EL datasets. Zhang Xiong [3] proposed a Multi-Feature Region Proposal Fusion Network (MF-RPN) structure for multi-scale defect detection. By extracting region proposals from different feature layers and combining them with a Multi-Scale Region Proposal Selection Strategy (MRPSS) to reduce redundancy, the model effectively improves its feature extraction ability for surface defects of different scales and types. Wang Yin [4] proposed the YOLO-WAD model based on YOLOv10n. The CSP bottleneck with two convolutions–wavelet transform convolution (C2f-WTConv) was used instead of C2f in the backbone network to expand the receptive field. ASF structure and new detection layer were introduced in the neck network. The CSP bottleneck with two convolutions–efficient multi-scale attention mechanism (C2f-EMA) and the Dynamic Head (DyHead) detection head was used to improve the detection ability.

The Attention Mechanism (AM) has shown significant advantages in photovoltaic panels defect detection. Through mechanisms such as feature reweighting, spatial domain focusing, and channel domain enhancement, it solves the problems faced in photovoltaic module defect detection, such as small object recognition, complex background interference, and multi-scale feature fusion. With the continuous improvement of detection accuracy and efficiency requirements in the photovoltaic industry, detection models based on AM have improved detection performance by selectively enhancing defect features and suppressing irrelevant background information. Su Binyi [5] innovatively proposed the Residual Channel Attention Gate Network (RCAG-Net) to solve the problem of feature disappearance in deep networks. This network achieves multi-scale feature fusion through RCAG modules. It uses global average pooling for feature dimensionality reduction and refinement, and generates attention maps using gating mechanisms to achieve channel feature reweighting. Combined with residual connections, it ensures seamless embedding of the module into the pre-trained model. Its excellent detection ability for small hotspot defects in photovoltaic power plants has been verified in practical detection systems. Qian Huimin [6] proposed an innovative segmentation before detection paradigm to solve the challenge of detecting small hotspots in infrared images. An efficient channel attention module was introduced into the object detection model developed based on YOLOv5s to eliminate aliasing effects in the feature fusion network. Multi-scale semantic information fusion was enhanced by adding a dedicated prediction head for small hotspots and improving the path aggregation network. Liang Qiang [7] proposed a multi-path feature weighted fusion method based on the YOLOv7 framework, which utilizes the channel dimension sensitivity of the Fusion Convolution Attention (FCA) module to enhance high-quality feature paths, reconstruct the backbone network and feature fusion structure, and introduce long residual edges to solve the singularity problem in the fusion of medium output tension. Liu Linjun [8] developed the CA-YOLOv5 network to solve the problem of diverse forms and small sizes of bird droppings defects. By embedding Coordinate Attention (CA) modules between the backbone and neck, while capturing channel relationships and position information, the feature extraction capability is enhanced. Additionally, a small object detection layer is added to achieve the fusion of shallow details and deep semantics, resulting in an average accuracy of 92%. This achieves a good balance between detection accuracy and model complexity. Zhu Jiahao [9] proposed the C2DEM-YOLO network to address the challenges of complex backgrounds and large-scale variations in EL images. The C2Dense module was designed to replace the original C2f module to enhance feature extraction capabilities, and the cross-space multi-scale attention was introduced for pixel level attention processing.

Attention mechanism and network structure optimization have become important methods to improve the performance of photovoltaic defect detection. The progress has been made in solving challenges such as weak defect features, complex backgrounds, and variable scales in electroluminescent. Liu Qing [10] broke through the limitations of traditional CNN in global information modeling by designing the Graph Channel Attention Module (GCAM). This module uses graph channel inference to generate feature representations that go beyond local receptive fields. Guo Shuqiang [11] integrated hollow space pyramid pooling and spatial attention mechanism in improving the feature extraction network of Mask R-CNN, using infrared video data captured by drones for end-to-end training. While maintaining an 8.2 FPS inference speed, the accuracy and recall of defect detection were increased to 89.8% and 84.4%, respectively. Gao Yihong [12] proposed a Partial Spatial Attention (PSA) mechanism to meet the deployment requirements of edge devices. It combines partial convolution with spatial attention to optimize the feature extraction process. Combined with a multi-channel feature fusion detection head and ShapeIoU localization method, mAP@0.5 of 87.2% and 72.0% were achieved on Panel-2 and Solar datasets, respectively, and low latency inference of 2.6 milliseconds was achieved on the Jetson Xavier NX platform. Cao Yukang [13] introduced attention mechanism and bidirectional feature pyramid network based on YOLOv5, and used GhostConv lightweight module and GELU activation function for model compression. On the EL image dataset, the accuracy was improved by 2% while the inference speed was increased by 20.3%, demonstrating the potential for fast and accurate detection in building photovoltaic systems. Zhou Peng [14] focuses on fine segmentation tasks and proposes a new Neighbor Attention Fusion (NAF) and a Multi-Level Pyramid Strategy (MLPS). Through pooling distribution mechanism, cross level feature interaction is promoted, and high-level attention is used to learn spatial neighborhood similarity. On self-built datasets and PSCDE datasets, 84.3% and 98.35% MIoU were obtained, respectively.

By deeply integrating machine vision and deep learning algorithms, the detection challenges in complex industrial environments have been effectively addressed. In the field of edge defect detection of photovoltaic glass, Xiong Jie [15] has developed a complete machine vision detection system, which adopts a high contrast imaging scheme with multiple light sources and a high stability transmission mechanism. Innovatively, dense residual units are integrated into the two parts of the Fire module in the SqueezeNet network to construct an improved model that can effectively extract key features of glass edge images and suppress interference factors such as water droplets. Ultimately, an average missed detection rate of 0.0064% and a false detection rate of 0.0075% were achieved, with a single detection time of only 2.715 s, fully meeting the dual requirements of precision and efficiency in industrial production. In response to the large differences in scale, diverse types, and complex backgrounds of defects in photovoltaic panels, Zhou Xinwen [16] proposed a LEM Detector based on Transformer architecture. This model introduces a linear feature enhancement module designed specifically for linear features to improve training performance, uses an efficient feature enhancement module to expand the receptive field and suppress redundant information, and uses a multi-scale and multi feature pyramid network to aggregate boundary and category information. It achieved a multi-scale defect detection accuracy of 94.7% on large-scale datasets. Faced with the domain adaptation challenge caused by data distribution bias in open world scenarios, Chen Haiyong [17] designed an aggregation allocation domain offset suppression network, which adopts a single domain generalization method completely independent of test samples. The module captures multi-scale contextual information to suppress background style shift, and the aggregation allocation module is used to achieve cross layer feature interaction learning. By combining normalized Wasserstein distance to reduce errors caused by boundary box position deviation, this model achieved high accuracy in testing scenarios of multiple production lines’ electroluminescence datasets.

In summary, although deep learning based visual inspection technology has made some achievements, it still faces the bottleneck problem of difficulty in balancing detection accuracy and computational efficiency, especially in identifying small defects such as cracks and broken grids on the surface of photovoltaic panels in complex backgrounds. This article proposes an AE-YOLO model based on the YOLOv11, which integrates the Adown and ECA. The model achieves synergistic optimization of performance and efficiency through dual path feature compression and channel adaptive weighting strategy. The Adown module utilizes a collaborative design of average pooling preprocessing and dual path convolution to reduce the computational burden of the model. The ECA enhances the response of defect sensitive feature channels through lightweight local cross channel interaction, improving the model’s ability to discriminate low contrast small defects. The experimental results show that AE-YOLO achieved good performance on the PVEL-AD dataset. mAP@0.5 reached 90.3%, the parameter count decreased by 18.7%, calculation decreased by 19%, and inference speed reached 259.56 FPS. The ablation experiment further verified the complementarity between Adown and ECA modules, providing an innovative solution for real-time and accurate defect detection of photovoltaic panels in industrial scenarios.

## 2. Materials and Methods

### 2.1. YOLOv11 Framework Structure

YOLOv11 (Version 11.0, Manufacturer: Ultralytics, Frederick, MD, USA) is a new-generation computer vision model. As the latest member of the YOLO series, it has achieved improvements in speed, accuracy, and functionality, making it suitable for complex tasks such as object detection, instance segmentation, and pose estimation.

YOLOv11 introduces the Cross Stage Partial with kernel size 2 (C3k2) in the backbone, which is the core component for efficient feature extraction. Its design combines parallel convolutional branches, variable convolution kernels, and lightweight strategies. It improves the detection accuracy and inference speed in complex scenes. It also introduces Cross Stage Partial with Pyramid Squeeze Attention (C2PSA), a key technical module for enhancing feature extraction. By combining the Cross Stage Partial (CSP) structure and Pyramid Squeeze Attention (PSA) mechanism, the multi-scale feature fusion capability and detection accuracy in complex scenes are improved. The decoupled detection head of YOLOv11 achieves a unity of lightweight and high precision through structural decoupling, depth-wise separable convolutions, and dual-label assignment.

### 2.2. Key Innovation Module

#### 2.2.1. Lightweight Dynamic Down-Sampling Module—Adown

Surface defects on photovoltaic panels usually have characteristics such as small size and subtle texture changes. Traditional down-sampling methods may lead to the loss of key features or difficulty in achieving a balance between computational efficiency and spatial resolution. For instance, standard convolutional down-sampling may introduce redundant calculations and increase ineffective computational overhead. The single pooling operations can overlook local small features and even lead to edge gradient dispersion. Other mixed down-sampling methods cannot fully utilize channel dimension information [18]. This paper selects the dual-path fusion lightweight dynamic down-sampling module Adown, which combines average pooling, maximum pooling, and dual-path convolution.

The improvement method is as follows. By performing average pooling preprocessing before feature segmentation, the smooth extraction of global features is enhanced. One branch retains the maximum pooling and convolution operation with the stride of 1, while the other branch only uses 3 × 3 convolutions to reduce the computational burden. This idea of using dual paths for feature processing is similar to the multi-layer aggregation idea of YOLOv9, which well balances high-frequency details and low-frequency background information. This module enhances the sensitivity to defects through a differentiated feature processing mechanism. By replacing some standard convolution modules in the original network structure with this module, the model’s computational and parameters are significantly reduced.

Input preprocessing:

(1) $$x=AvgPool(x_{in})$$

Channel segmentation:

(2) $$x_{1},x_{2}=chunk(x;\dim=1)$$

Branch 1:

(3) $$y_{1}=Conv_{3\times 3}(x_{1};s=2)$$

Branch 2:

(4) $$y_{2}=Conv_{1\times 1}(MaxPool(x_{2};k=3,s=2))$$

Feature fusion:

(5) $$y=Concat(y_{1},y_{2};\dim=1)$$

**Figure 1:**
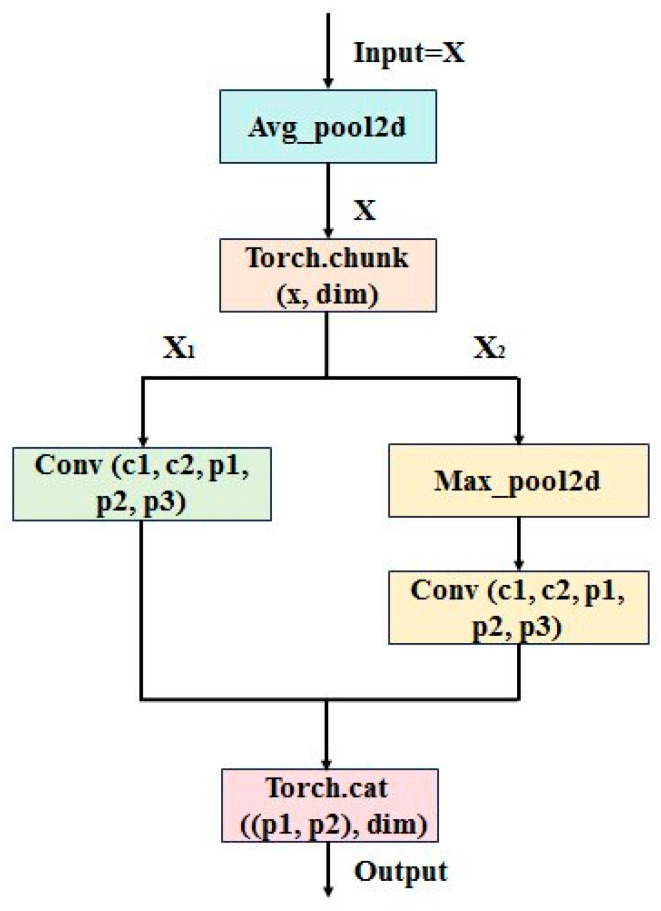
Adown structure.

The calculation process is shown in Figure 1.

The specific process of processing and feature fusion of the feature image using the Adown module is as follows.

**Figure 2:**
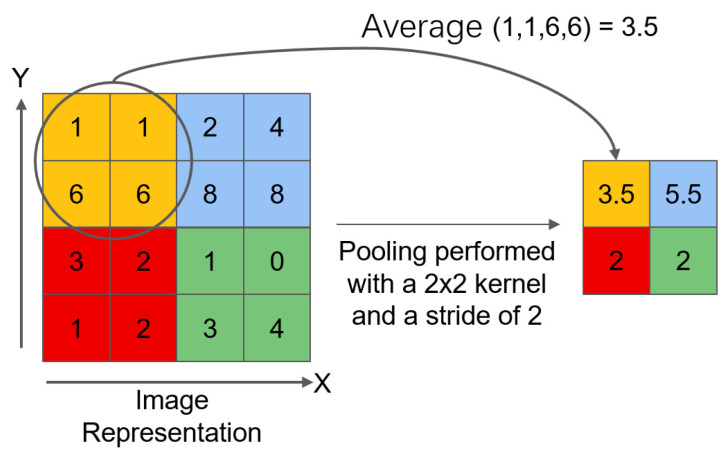
Average pooling.

Step 1: Average pooling preprocessing. Firstly, the input feature segmentation map undergoes average pooling preprocessing. Average pooling operates by calculating the mean of elements within the pooling window to process the input feature map, with the core purpose of spatial down-sampling while keeping the number of channels unchanged. The average pooling operation uses a 2 × 2 pooling window, with a window movement stride set to 1, and no padding is performed. When calculating the output size, rounding down is used, and padding values are included in the mean calculation process. The process of average pooling is shown in Figure 2. The above operations achieve a halving of size in the spatial dimension, while the number of channels remains unchanged. Its purpose is to reduce data volume, decrease computational complexity, and improve network efficiency. Meanwhile, it retains the overall information of the input features to some extent, avoiding information loss due to excessive down-sampling, and providing suitable input for subsequent feature extraction and target detection.

Step 2: Channel dimension segmentation. After completing the average pooling preprocessing, the channel dimension is split. The feature map is evenly divided into two tensors along the channel dimension (dim = 1), halving the number of channels in the feature map, thus decreasing the subsequent computational load and improving processing speed. It also allows for the extraction of different feature information, creating conditions for subsequent parallel processing. From a computational resource perspective, decreasing the number of channels means that the number of parameters and computational load correspondingly decrease in operations like convolution, effectively lightening the training and inference burden of the model. Different sub-tensors can focus on extracting different types of features. For instance, some sub-tensors may be more adept at extracting texture features, while others may be more sensitive to shape features. This parallel extraction of different features helps to improve the network’s ability to detect complex targets and enhance generalization performance.

Step 3: Branch feature processing. After completing the channel dimension segmentation, different feature processing operations are performed on the two sub-tensors obtained from the segmentation, forming two independent processing branches.

In the first branch processes, feature transformation is carried out through convolutional layer. It uses the 3 × 3 convolution kernel, stride of 2, and padding of 1. The convolution kernel can capture the feature information of local regions. The stride of 2 achieves down-sampling of the feature map, halving the size of the output feature map. The padding of 1 helps to maintain the boundary information of the feature map, preventing the loss of boundary features due to down-sampling. This operation reduces the size of the feature map while increasing the level of feature abstraction.

**Figure 3:**
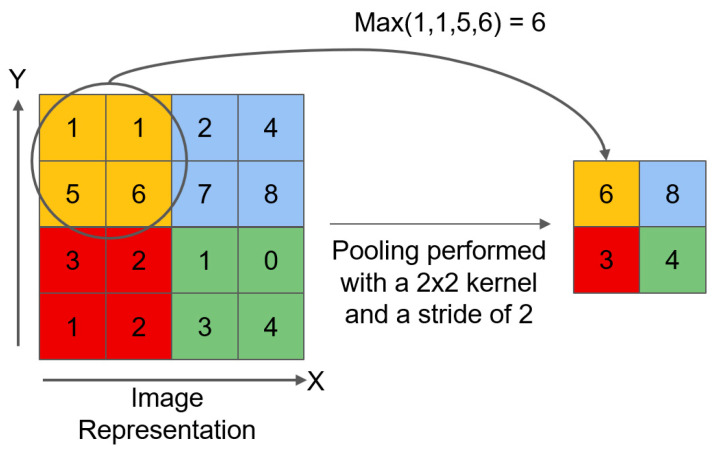
Maximum pooling.

The second branch also employs convolutional layers for feature manipulation, but unlike the first branch, the convolutional layers use the 1 × 1 convolution kernel with a stride of 1 and padding of 0. The 1 × 1 convolution plays a unique role in neural networks. It is used for adjusting the number of channels in feature maps and performing linear combinations of features. The 1 × 1 convolution kernel does not change the spatial dimensions of the feature map. Because the stride is 1 and no padding is performed, its main function is to adjust the number of channels in the feature map to achieve linear transformation of features. After the convolution operation, a two-dimensional maximum pooling operation is performed on the segmented sub-tensor, using 3 × 3 pooling window, stride of 2, and padding of 1. The process of maximum pooling is shown in Figure 3. It will help the network extract more representative features, improve sensitivity to target features, and thus enhance the performance of object detection.

Step 4: Feature fusion. After the feature processing of the two branches, it is necessary to perform feature fusion on them. In this step, the processed sub-tensors ×1 and ×2 are concatenated along the channel dimension using “torch.cat” command (dim = 1 indicates concatenation along the channel dimension), and the concatenated result is returned. (×1, ×2) are the tensors to be concatenated, merging the different features extracted from the two branches, and restoring the number of channels of the original feature map. This extracts diverse features, allowing the network to comprehensively utilize various feature information, thus more accurately detecting the position and category of the target object. Features extracted from different branches may describe the target object from different perspectives. Through feature fusion, these complementary features can be integrated to form a more comprehensive and discriminative feature representation, providing strong support for subsequent target detection tasks.

Therefore, based on the Adown module, efficient processing and feature fusion of the input feature map are achieved through four closely connected steps: average pooling preprocessing, channel dimension splitting, branch feature processing, and feature fusion.

#### 2.2.2. Efficient Channel Attention

The AM is a method that mimics the human visual and cognitive system, allowing neural networks to focus on relevant parts when processing input data. By using AM, neural networks can learn and selectively pay attention to important information in the input. It improves the model’s performance and generalization ability. The AM originates from the human visual system. When humans observe external objects, they do not view the objects as a whole, but tend to selectively acquire certain parts of the observed objects based on needs. When observing a person, one forms an overall impression by first observing the person’s face and then combining information from different parts of the body. Therefore, it has drawn much attention due to its powerful modeling capabilities. It can focus on the most relevant information, thereby improving the performance of the model.

The core idea of the AM is to weigh the information of different parts according to the relevance of the input. The formula is shown as Equation (6).

(6) $$Attention(Q_{uery},K_{ey},)=soft\max\left(\frac{Q_{uery},K_{ey}^{T}}{\sqrt{d_{Key}}}\right)V_{alue}$$

In the equation, *Q_uery_*, *K_ey_*, and *V_alue_* are the linear transformations of the input vectors. $\sqrt{d_{Key}}$ is the dimension of the *K_ey_* vector.

A typical attention model consists of the following four parts: Input embedding layer, which converts discrete input data into continuous vector representations. The attention calculation layer, which is the core part of the model, determines the weighting method of the value vector by calculating the similarity between the query and the key. Feed-forward neural network are usually followed by a feed forward neural network after the multi-head attention layer. Residual connections and layer normalization are used to solve the problem of gradient vanishing and can effectively improve convergence speed and stability.

However, traditional channel AM, such as Squeeze-and-Excitation (SE), learn channel dependency through Fully Connected layers (FC). The large number of parameters and computational complexity in the FC layers limit the lightweight characteristics of the model. It can lead to redundant calculations and low parameter efficiency, and may result in the loss of critical information.

The ECA mechanism is a lightweight channel attention module that avoids establishing fully connected operations by calculating the convolution kernel channel by channel. It does not require excessive parameters but can better the performance of the model, finding a balance between computational complexity and accuracy improvement [19]. Moreover, it has higher efficiency and better performance compared to other attention modules.

**Figure 4:**
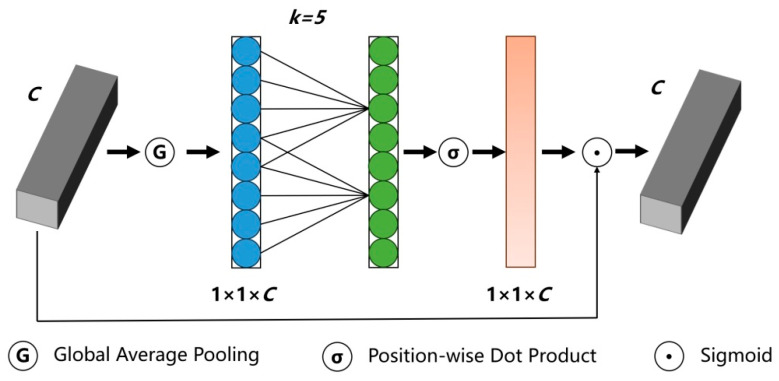
The ECA structure.

The ECA structure is shown in Figure 4.

The ECA includes the following three core steps.

Global Average Pooling (GAP):

(7) $$y_{gap}=AdaptiveAvgPool2d(x)$$

Convolutional Cross-Channel Interaction:

(8) $$y_{Conv}=Conv1d(y_{gap};k)$$

(9) $$k=\frac{\log(C)}{\gamma }+b$$

Here, *γ* and *b* are hyper-parameters.

Sigmoid activation:

(10) $$y_{att}=Sigmoid(y_{Conv})$$

The specific process is as follows.

**Step 1:** Adaptive average pooling. The feature map is the result of the network performing multiple convolution operations on the input image, containing rich spatial and channel information. However, in some cases, excessive spatial details may interfere with the model’s grasp of the overall features.

The main task of the adaptive average pooling layer is to compress the spatial dimensions of the input feature map to 1 × 1. Assuming the input feature map has a shape of H × W × C, where H represents height, W represents width, and C represents the number of channels. Through the adaptive average pooling operation, it will obtain an output with the shape of 1 × 1 × C. Thus, a global spatial information descriptor was obtained, which represents the global average pooling value for each channel. Global spatial information was also extracted, allowing the model to understand the feature distribution of each channel at a macro level, providing a foundation for subsequent channel attention calculation.

**Step 2:** Generate channel attention weights. After obtaining the global spatial information descriptor, it is necessary to determine the importance of each channel, which is to generate channel attention weights. This paper accomplishes this task by setting up a one-dimensional convolutional layer. The input and output channels are both set to 1, and the convolution kernel is 3 × 3, ensuring that the output length after convolution is consistent with the input length. During the convolution operation, the one-dimensional convolutional layer performs local feature extraction on the input global spatial information descriptor along the channel dimension. Through the convolution operation of the convolution kernel and the input data, information interaction and processing between channels are realized. To convert the output of the convolution operation into effective channel attention weights, a sigmoid activation function is adopted. By processing with the activation function, the attention weights of each channel are obtained, which in turn reflects the importance of each channel in the feature map. The closer the weight value is to 1, the more important the feature of the channel. The closer the weight value is to 0, the less important the feature of the channel.

**Step 3:** Enhance the features of important channels, and suppress the features of unimportant channels. After obtaining the channel attention weights, it is necessary to multiply the input feature map and channel attention weights element by element. Then, each channel of the input feature map is dynamically adjusted by the size of the channel attention weights. For important channels, their corresponding attention weights are larger, and the feature values of these channels will be enhanced after multiplication, thus being more prominently focused on by the model in subsequent processing. For unimportant channels, their corresponding attention weights are smaller, and the feature values of these channels will be suppressed after multiplication, reducing the interference of these channels on the model’s decision-making.

In summary, the ECA achieves adaptive calibration of feature channel information through three core steps, namely, adaptive average pooling, one-dimensional convolution with sigmoid activation, and channel by channel weighting.

## 3. Experimental Results and Analysis

### 3.1. Dataset and Evaluation Metrics

The PVEL-AD [20,21,22] is the largest-scale benchmark dataset for photovoltaic battery defect detection globally, jointly released by Hebei University of Technology and Beijing University of Aeronautics and Astronautics, aiming to provide a standardized evaluation platform for photovoltaic defect detection algorithms. This dataset is constructed based on electroluminescence (EL) imaging technology, covering various complex scenarios and defect types, containing over 30,000 images, of which 4500 are annotated. This paper selects seven types of defects for experimentation: crack, finger, black core, thick line, star crack, printing error, and short circuit. The paper divides 3600 images into training sets and 900 images into validation sets in a 4:1 ratio.

**Figure 5:**
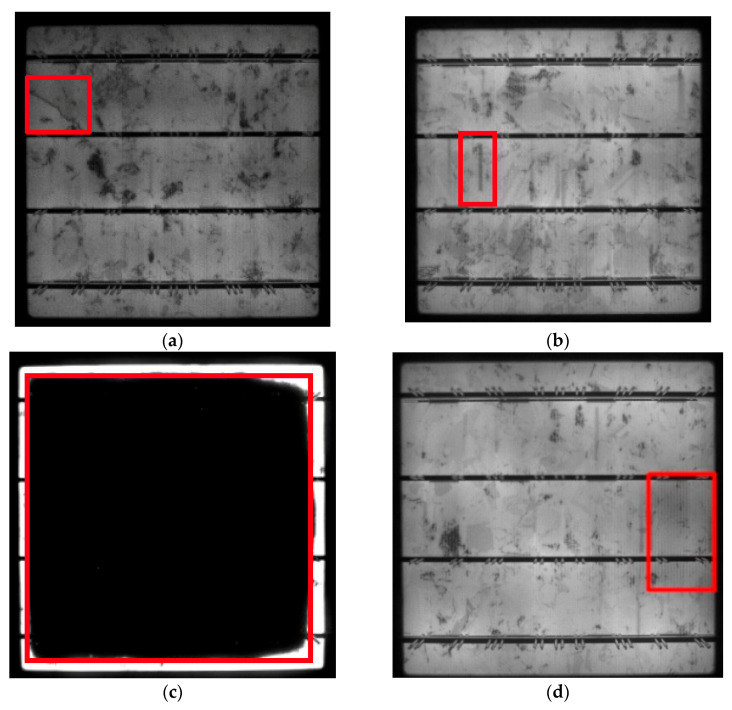

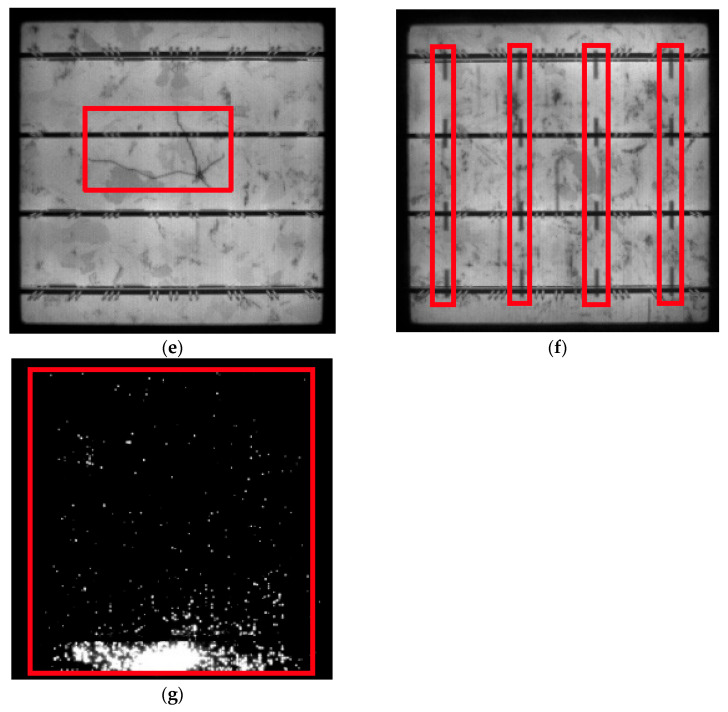
Dataset samples. (**a**) Crack. (**b**) Finger. (**c**) Black core. (**d**) Thick line. (**e**) Star crack. (**f**) Printing error. (**g**) Short circuit. The detailed introduction of the above seven types of defects is as follows.

The dataset for surface defect samples is shown in Figure 5. To clearly locate the defect area, red wireframes have been used in the figure to label the specific locations of defects in each sample, in order to visually distinguish the morphological differences between defects and normal areas.

1. Crack

The crack refers to fractures occurring in the silicon material inside the solar cell. The cracks detected in this article are mainly visible cracks, which are visible to the naked eye. The occurrence of cracks is often related to improper operation during production processes, such as improper force application during handling, welding, and lamination. The commonly used method for detecting cracks is EL detection. Areas with cracks cannot emit light, and will appear as clear black lines in the EL image.

2. Finger

The finger refers specifically to abnormalities occurring in the thin grid lines, resembling fingers, on the front side of the solar cell panel used for current collection. This type of defect manifests as broken, missing, or uneven grid lines. These defects are primarily caused by issues during the screen printing process, such as blocked screen plates, uneven squeegee pressure, or improper paste viscosity. It leads to discontinuous printing of the grid lines. This defect can be detected through EL inspection, where the broken grid areas will exhibit local dark areas.

3. Black Core

The black core, also known as black heart, manifests in EL images as an irregular, approximately circular dark area. The main reason is that the lifetime of minority carriers in the central zone of the cell is much shorter than that in the edge region.

4. Thick line

The thick line refers to the cell busbar, especially the main busbar or fine busbar, width exceeding the process specification and becoming too wide. The main cause of this defect is improper printing parameters, such as too little squeegee pressure, improper snap-off distance between the screen and the silicon wafer, etc. In addition, wear or damage to the screen busbar pattern can also lead to thick line defects, which manifest as obvious uneven thickness.

5. Star Crack

The star crack is a special form of cracks, typically radiating out from a central point, resembling a star or spider web. The main cause is the impact of point-like external forces on the solar cell during production or transportation, such as small protrusions on equipment or collisions with hard objects. When a local point is subjected to a large external force, this force will be released to the surrounding area, causing the silicon wafer to crack with that point as the center. Under EL detection, star crack will exhibit typical radial black patterns, and severe star cracks can be visible to the naked eye.

6. Printing Error

The printing error constitutes a broad category, referring to issues other than finger defects and thick lines in the screen printing process. These mainly include printing offsets, incomplete printing of back electrodes, and smudges. In EL inspection, printing errors can lead to uneven brightness and darkness in EL images.

7. Short Circuit

The short circuit refers to internal leakage in the solar cell, where the P-N junction is partially damaged, causing the current to be bypassed and unable to flow through the external circuit to perform work. In EL images, short circuits manifest as local or overall abnormal dark areas.

The above-mentioned defects are the focus of monitoring and prevention in the photovoltaic manufacturing industry. This article uses deep learning techniques to identify defective solar cells, ensuring the quality and long-term power generation reliability of the final photovoltaic modules.

Intersection over Union (IoU) is an algorithm that calculates the overlapping ratio between different images, primarily used for object detection. A higher IoU value indicates that the predicted box overlaps more with the real box. Therefore, its detection and positioning are more accurate. The calculation formula is shown in Equation (11).

(11) $$IoU=\frac{A\cap B}{A\cup B}$$

The precision is the proportion of truly positive samples among those predicted as positive, and its calculation formula is shown in Equation (12).

(12) $$P=\frac{TP}{TP+FP}$$

The recall rate reflects the proportion of all true positive samples that were successfully predicted, as shown in Equation (13).

(13) $$R=\frac{TP}{TP+FN}$$

The Precision Recall (PR) curve is the evaluation of the performance in predicting positive categories. It takes accuracy as the vertical axis and recall rate as the horizontal axis, which is suitable for imbalanced category scenarios.

The F1-Score is the harmonic mean of precision and recall, and its calculation formula is shown in Equation (14).

(14) $$F1=2\frac{\left(P\times R\right)}{P+R}$$

Mean Average Precision (mAP) is used to measure the performance of the target detection algorithm. It is obtained by the comprehensive weighted average of the Average Precision (AP) of all kinds of detection.

(15) $$mAP=\frac{1}{n}\sum_{i=0}^{n}AP_{i}$$

Here, AP averages all categories of Area Under the Curve (AUC), and the average accuracy is shown in Equation (16).

At the same time, mean Average Precision at IoU threshold 0.50 (mAP@0.5) is calculated as the average precision under different IoU thresholds, followed by taking the category average. Therefore, mAP@0.5 represents the average when the threshold is 0.5.

(16) $$AP=\frac{1}{n}\sum_{i=0}^{n}AUC_{i}$$

The confusion matrix is a table used to visually display the comparison between the prediction results of a classification model and the true labels. By using the confusion matrix, one can intuitively see the performance of the model in each category.

### 3.2. Implementation Details

The proposed AE-YOLO evolved from the YOLOv11 baseline model. The original YOLOv11 model still has the issue of high computational demand, making it difficult to meet the requirements of real-time monitoring in industrial scenarios or deployment on edge devices. By modifying some convolution modules in the backbone with Adown modules significantly reduces the number of parameters and computational demand, enhancing the model’s adaptability to devices.

**Figure 6:**
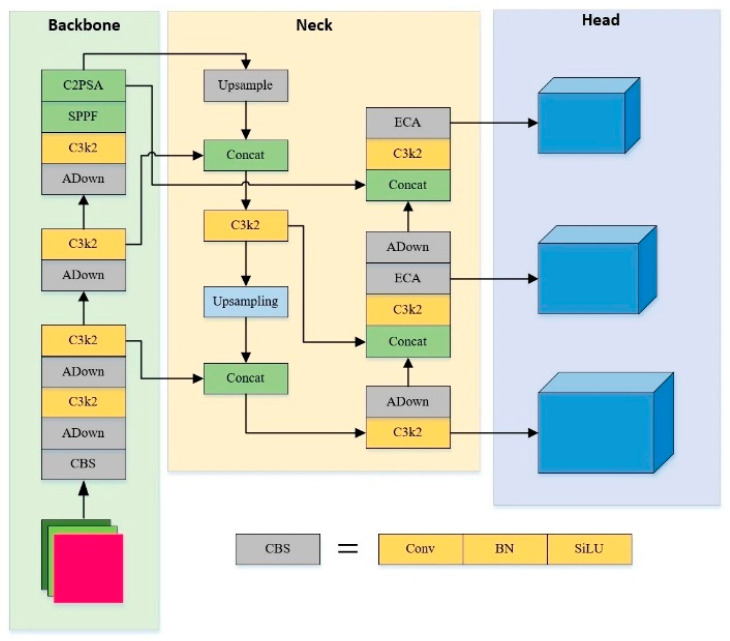
AE-YOLO network structure.

The YOLOv11 has insufficient multi-scale defect perception, especially for defects like crack, which are usually small and elongated with low contrast to the background. Additionally, the images in the EL dataset contain a significant amount of noise. This will interfere with the learning of the model and reduce its feature extraction capability, leading to missed detection. By adding ECA to the large and medium-sized detection layers, the recognition success rate for crack types can be improved. AE-YOLO network structure is shown in Figure 6.

The figure indicates the specific location where the Adown module replaces the original standard convolution for down-sampling in the network. Adown is applied to the key step of feature map scale reduction in Backbone, demonstrating intuitively how it achieves efficient down-sampling through lightweight dual path design. Meanwhile, the structural diagram indicates that the ECA mechanism is integrated into the medium and large detection layers of the Neck. This figure illustrates the data flow and hierarchical interaction relationships between Adown, ECA, and YOLOv11 basic components (C3k2, SPPF, C2PSA, etc.). It shows how the features down-sampled by Adown flow into subsequent layers, and how the ECA module enhances the fusion feature in the detection path of medium to large object. This fully embodies the collaborative optimization idea of Adown providing high-density feature input and ECA performing channel attention calibration on this basis. The YOLOv11 model is used as the core architecture to carry out improvement research. Table 1 is the experimental environment. This configuration fully guarantees the demand for computing power resources in the model training stage. This framework ensures compatibility between mainstream deep learning frameworks and hardware drivers. It provides a stable and reliable foundation for the reproducibility of the experiment and the consistency of the results.

### 3.3. Experiment

The setting of the main hyperparameters is carried out under a unified framework. In terms of basic training, the training period (epoch) is set to 100 to ensure that the model fully converges. The batch size is set to 16. The input images size is 640 × 640 pixels, retaining sufficient defect detail features. The optimizer uses Stochastic Gradient Descent (SGD) with momentum set to 0.937 to accelerate convergence and suppress oscillations. The weight decay coefficient is 0.0005, and L2 regularization is applied to prevent over-fitting. The learning rate (lr) adopts a linear decay strategy, with an initial learning rate of 0.01 and a final decay to 0.0001. Before training, three learning rate warm-up cycles are enabled. During this period, the momentum and bias learning rates are set to 0.8 and 0.1, respectively, to achieve stable initialization in the early stages of training.

**Figure 7:**
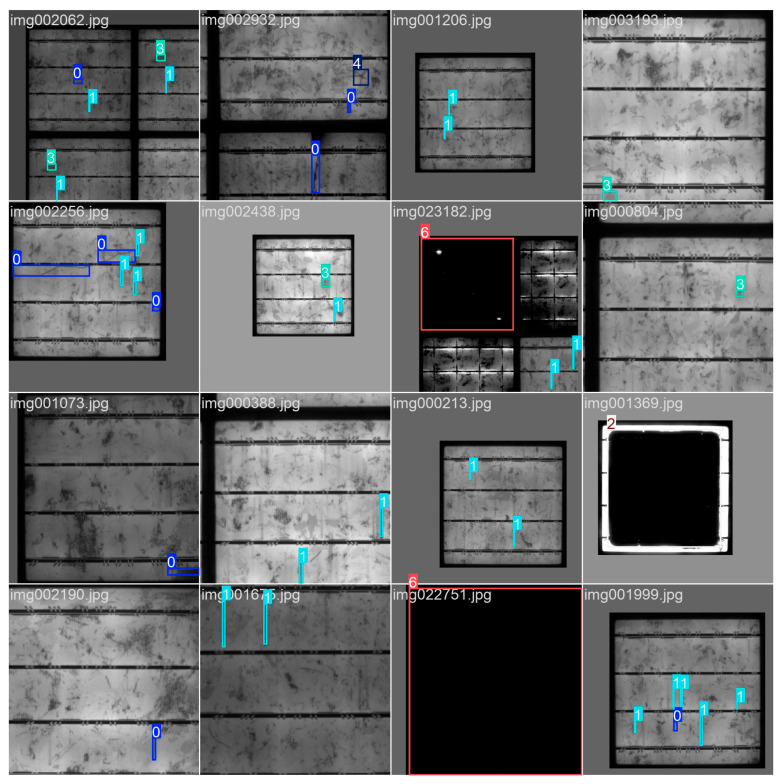
Enhanced dataset.

In response to the multi-tasking characteristics of object detection, fine adjustments are made to the weight of the loss function. The bounding box regression loss gain is set to 7.5. The classification loss (cls) is set to 0.5, and the distribution focus loss (dfl) gain is set to 1.5 to optimize the probability distribution modeling of the bounding box. The data augmentation adopts a comprehensive approach, with color space perturbations covering hue, saturation, and brightness, geometric transformations including translation and scaling, and horizontal flipping with a probability of 0.5. Meantime, to enhance the model’s generalization ability and detection accuracy, four training images are spliced into one, simulating complex scenes, reducing the possibility of over-fitting, and enhancing the model’s generalization ability in complex environments, as well as strengthening the detection effect on small and occluded targets. Figure 7 is the enhanced dataset. Mosaic enhancement is turned off in the last 10 rounds of model training. In the inference and validation phase, the IoU threshold for non-maximum suppression is set to 0.7, and automatic mixed precision training is enabled to improve training speed and control graphics memory usage. In Figure 7, the numbers 0 to 6 and their corresponding boxes are used to identify and distinguish different types of defects. Their specific meanings are: 0 represents crack, 1 represents finger, 2 represents back core, 3 represents thick line, 4 represents star crack, 5 represents printing error, and 6 represents short circuit.

**Figure 8:**
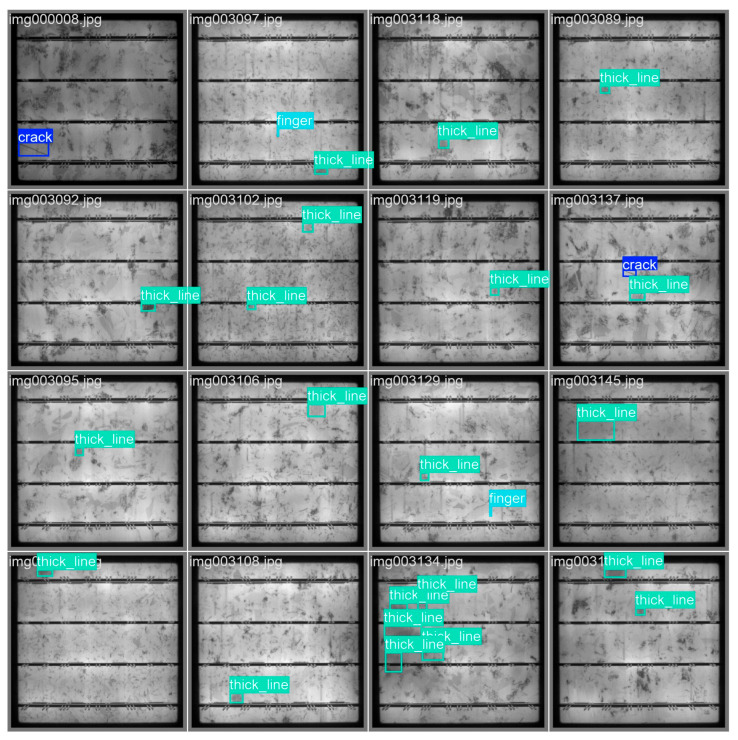
Label.

Figure 8 shows the label images file after training. The images show the visual images related to labels in the data set and give examples of images with information such as the location and category names of different categories of targets, such as crack, finger, and thick line. It is used to visually present the labeling situation in the data set and help users quickly understand the content and style of labeling.

**Figure 9:**
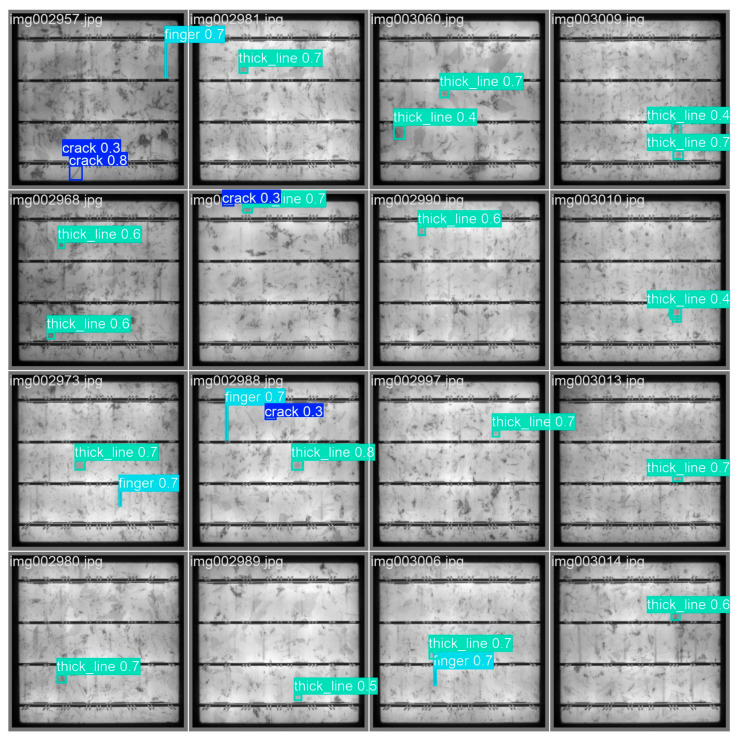
Test results.

Figure 9 is the image of the test results. After testing by the AE-YOLO model, many surface defects were found. These defects are marked in the image as bounding boxes and each bounding box corresponds to a specific defect type, location, and confidence score.

The AE-YOLO model exhibits excellent detection performance in dense defect areas. It not only achieves precise defect localization, but also outputs correct category labels and high confidence, verifying its excellent practical performance.

**Figure 10:**
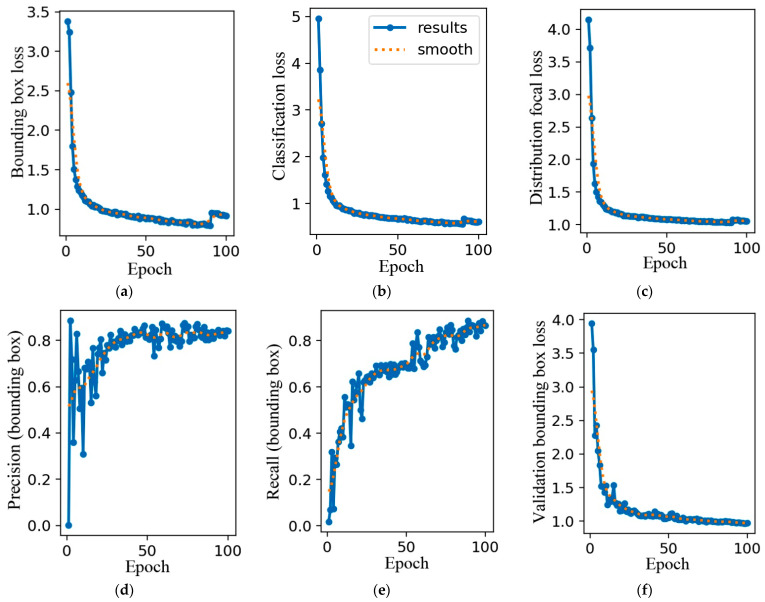

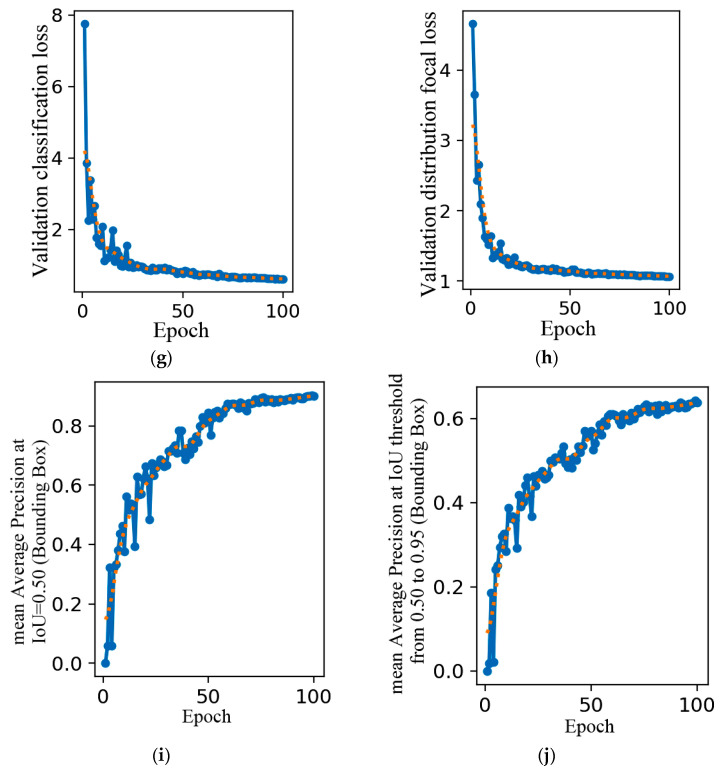
Result of training. (**a**) Train/Bounding box loss. (**b**) Train/Classification loss. (**c**) Train/Distribution focal loss. (**d**) Train/Precision (bounding box). (**e**) Train/Recall (bounding box). (**f**) Train/Validation bounding box loss. (**g**) Train/Validation classification loss. (**h**) Train/Validation distribution focal loss. (**i**) Train/mean Average Precision at IoU=0.50 (Bounding Box). (**j**) Train/mean Average Precision at IoU threshold from 0.50 to 0.95 (Bounding Box). Note: (1) The blue line (results) represents the raw values of the indicators directly calculated after each training batch (Epoch). It reflects the true changes in various performance indicators of the model during the training process. (2) The orange dashed line (smooth) is the curve obtained by applying a smoothing algorithm to the blue raw data. It reflects the overall trend of changes in the training metrics of the model.

The training loss and evaluation metrics of AE-YOLO model are shown in Figure 10, which are used to analyze its convergence and detection performance.

In Figure 10a, the initial bounding box loss is about 3.5, which shows a rapid downward trend during training iterations and ultimately stabilizes in the low loss range close to 0.5. It indicates that the model can reduce the spatial deviation between the predicted box and the real defect area. The boundary fitting ability for irregular shaped defects such as crack and thick line is constantly improving. Figure 10b reflects the classification accuracy of the model for various defects, such as crack, black core, and short circuit. The initial loss is about 5, which rapidly decreases and tends to stabilize during the training process, eventually approaching 0. This indicates that the model can learn the feature differences in different defects. When facing the complex background, the confusion in classifying various types of defects is reduced, and the confidence and accuracy of classification are continuously optimized. It reflects the increasing ability of the model to distinguish multiple types of defects. Figure 10c is an indicator used to measure the model’s ability to handle class imbalance when classifying each prediction box into multiple categories. The initial loss is about 4.0, which shows a significant downward trend with training iterations and eventually stabilizes below 1.0. It indicates that the model can grasp the distribution characteristics of the bounding box when dealing with the multi-scale distribution of defects. This reduces prediction fluctuations caused by differences in defect scale, further enhancing the detection robustness of the model in complex scenarios.

In the initial stage, the precision curve fluctuates and the value is relatively low in Figure 10d. It is because at the beginning of training, the parameters are initialized randomly, and its ability to extract features from data and classify them is weak. The model incorrectly predicts many background areas as targets, resulting in the low accuracy. Then, it shows an overall upward trend, but there are still fluctuations. As training progresses, the model begins to learn some basic features of surface defects and gradually distinguishes between targets and backgrounds. However, due to the potential complexity and noise in the training data, the model performs unstably on certain batches of data, causing fluctuations. In the later training stage, it tends to stabilize, with the value remaining at a relatively high level, close to 0.8. At this point, the model has fully learned the features in the data and can accurately determine whether a target exists. It can identify various complex defects, such as crack, finger, and star crack, and the number of false positive samples has been reduced, thus maintaining a high and stable precision.

From Figure 10e, it can be seen that the recall rate is very low in the initial stage. When the model starts training, its ability to recognize the target is limited, and many real surface defects cannot be detected, resulting in a large number of false negative samples. In the middle training stage, the recall rate rises rapidly. As the model continues to learn, it can recognize more surface defect features, and its detection ability gradually increases. The model starts to detect some previously missed surface defects, and the number of false negative samples decreases. At last, the recall rate growth slows down and tends to stabilize, increasing to above 0.9, enabling more comprehensive detection of various surface defects. The model has achieved high accuracy in detecting the target, but there may still be some extreme cases where the target cannot be detected, such as thick line and crack.

Figure 10f is used to measure the accuracy of the model in locating the boundary boxes of photovoltaic panel defects on the validation set. The initial loss value is about 4.0, which rapidly decreases during training iterations and eventually stabilizes within the range of 1.0–1.5. This indicates that it can fit its true bounding box on the validation set. The generalization ability of bounding box localization has been validated, providing support for accurate spatial localization of defects. Figure 10g reflects the accuracy of the model in classifying various types of defects on the validation set. The initial loss is about 8.0, then rapidly decreases and tends to stabilize. It can stably distinguish the differences between various types of defects. The consistency in confidence and accuracy in distinguishing defect categories on the validation set reflects the model’s generalization ability in multi class defect classification tasks. Figure 10h is used to measure the accuracy of the model’s predicted bounding box position distribution, which focuses on the positioning accuracy. Its initial loss is approximately 4.5, which significantly decreases with training iterations and eventually stabilizes below 1. There are significant scale differences in defects of photovoltaic panels, ranging from small fine cracks to larger thick line, with complex distribution characteristics. The stable decrease in this loss indicates that it can grasp the distribution of bounding boxes of defects at different scales on the validation set. Even when faced with untrained defect scale samples, it can reduce prediction fluctuations and verify the model’s generalization robustness in multi-scale defect detection scenarios.

Regarding Figure 10i, in the initial stage, the mAP@0.5 is relatively low. Due to the poor matching between the predicted bounding boxes and the true boxes, many predicted boxes have the IoU with the true boxes less than 0.5, resulting in a lower average precision. In the middle training stage, mAP@0.5 increases significantly. As the model learns, the matching between predicted boxes and true boxes gradually improves. It can locate the position and range of surface defects, enabling more predicted boxes to have the IoU with the true boxes exceeding 0.5. At last, mAP@0.5 tends to stabilize and eventually reaches 0.903. At this point, it has achieved good detection performance with the IoU threshold of 0.5. The model can stably detect and locate various surface defects, including small-sized defects such as thick line and star crack.

Regarding Figure 10j, in the initial stage, the mAP@0.5:0.95 is very low. This is because the metric considers a higher IoU threshold, which demands higher detection accuracy from the model. At the beginning of training, the IoU is low, so the average precision is at a low level. In the middle training stage, mAP@0.5:0.95 gradually increases, but the rate of increase is relatively slow. As the model learns, the degree of matching between the predicted boxes and the true boxes continues to improve, but it is still difficult to meet the higher IoU threshold. The model needs to locate surface defects more accurately and predict the shape and position of defects more precisely. In the later training stage, mAP@0.5:0.95 tends to stabilize and exceeds 0.6. At this point, the model can maintain a certain detection performance under different IoU thresholds and accurately identify the above seven types of defects.

**Figure 11:**
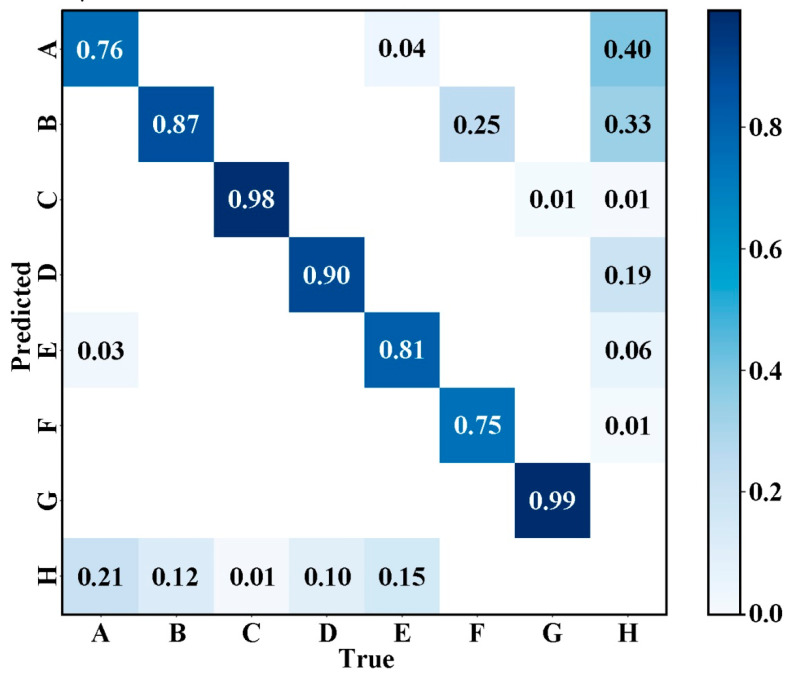
Normalized confusion matrix. A—crack, B—finger, C—black core, D—thick line, E—star crack, F—printing error, G—short circuit, and H—background.

Figure 11 shows the normalized confusion matrix of the AE-YOLO model on the PVEL-AD dataset. It used to visually evaluate the classification performance of the model for various types of defects. The horizontal axis represents the true defect category, and the vertical axis represents the model predicted category. The value of each cell represents the normalized proportion of the predicted results for that category, with dark blue indicating a high proportion and light colors indicating a low proportion. From the main diagonal, the model has a relatively high overall accuracy in identifying various types of defects. For example, the accuracy of category C is 0.98, category G is 0.99, the accuracy of categories B, D, E, and F are also 0.87, 0.90, 0.81, and 0.75, respectively, and category A is 0.76. Therefore, the model has strong discriminative ability for most defect categories. The values of non-main diagonals reflect the confusion of categories. The values above the main diagonal indicate the proportion of misclassifications where the true category is a certain category but is predicted as another category. For example, the proportion of true E being misclassified as A is 0.04, the proportion of true F being misclassified as B is 0.25, and so on. These values reflect the degree of confusion between different defect categories in the model, with smaller values indicating better category discrimination. The value below the main diagonal indicates the proportion of missed detections where the true category is a certain category but is mistakenly classified as H. The proportion of true A being misjudged as H is 0.21, the proportion of true B being misjudged as H is 0.12, etc. These values reflect the missed detection risk of various types of defects in the model, and the smaller the value, the more reliable the model’s identification of such defects. Overall, most defect categories can be identified and category confusion can be effectively controlled, indicating the robustness and reliability of the model in detecting defects under multi-scale and complex backgrounds.

In the experimental environment of this paper, relevant experiments are conducted based on the PyTorch (Version: 2024.3.4, Community Edition, Manufacturer: JetBrains s.r.o., Prague, Czech Republic) deep learning framework. After 100 iterations of training, AE-YOLO and all comparison methods obtained the following experimental results. The results are shown in Table 2.

Through the analysis of experimental data, the following main conclusions can be drawn. (1) mAP@0.5: The YOLO series models overall performed the best, with YOLOv5s and YOLOv11 significantly outperforming other models. SSD performed decently with smaller input sizes but still lagged behind the YOLO series. In contrast, the accuracy of Faster R-CNN was significantly lower, as cracks often appear as slender, curved, or irregular geometric shapes, and Faster R-CNN relies on rectangular bounding boxes for localization, which makes it difficult to precisely fit such targets, leading to large IoU calculation errors. Cracks have a wide range of length and width variations, requiring the model to have multi-scale perception capabilities, but fixed-size anchor boxes cannot effectively cover, resulting in low-quality candidate regions generated by the Region Proposal Network (RPN). The redundancy of two-stage object detection deserves attention in object detection tasks. EfficientDet, as a general object detection model, is lightweight and efficient, extracting high-level semantic features through multiple down-sampling operations. (2) Computational efficiency: EfficientDet had the lowest computational demand but sacrificed accuracy. YOLOv11 and AE-YOLO maintained a high mAP@0.5 (0.892~0.903) with moderate computational demand, demonstrating a better balance. Meantime, the AE-YOLO has a computational complexity of 5.3 GFLOPs, significantly lower than the original YOLOv11′s 6.4 GFLOPs, which theoretically confirms the effectiveness of its lightweight design. Faster R-CNN, despite having extremely high computational demand (370.26G FLOPs), had the worst performance, indicating its low resource utilization efficiency. (3) Parameter Efficiency: The AE-YOLO has the fewest parameters, only 2.1M, which is an 18.9% reduction compared to the standard YOLOv11 model. (4) mAP@0.5:0.95: The AE-YOLO model achieved the mAP@0.5:0.95 value of 0.63813 on the PVEL-AD dataset. It is not only better than the original YOLOv11n model’s 0.63422, but also significantly higher than YOLOv5s’ 0.60481, YOLOv7 tiny’s 0.5782, and traditional architectures such as SSD and Faster R-CNN. This result fully validates the effectiveness of the collaboration between the Adown module and the ECA mechanism. (5)Inference speeds (ms/frame): When the input size is 640 × 640 pixels, the AE-YOLO model achieves an inference speed of 259.56 FPS, which is slightly lower than the original YOLOv11 (297.98 FPS), but still significantly higher than other comparison models (Faster R-CNN’s 52.10 FPS, SSD’s 97.20 FPS, etc.), fully meeting the frame rate requirements for industrial real-time detection. The slight decrease in inference speed is mainly due to the additional computational path introduced by the ECA, but its gains in accuracy (mAP@0.5 improved to 90.3%) and feature discrimination ability are more significant. (6) Energy efficiency (mAP@0.5/Watt): The energy efficiency of AE-YOLO model is 0.0367, which is basically the same as the original YOLOv11′s 0.0371, indicating that the energy efficiency level is maintained while significantly improving the detection accuracy. Horizontal comparison shows that the energy efficiency performance of AE-YOLO is significantly better than traditional architectures such as Faster R-CNN, SSD, and EfficientDet. Although YOLOv7 tiny has a higher energy efficiency value of 0.0779, its detection accuracy is 4.4% lower than AE-YOLO. This comparison highlights the good balance achieved by AE-YOLO between accuracy and energy efficiency. The energy efficiency analysis further validated the effectiveness of the improvement strategy. The Adown reduces computational overhead by optimizing the feature compression process, while the ECA achieves feature calibration at minimal cost. The synergistic effect of the two improves performance while maintaining energy efficiency competitiveness.

To explore the performance characteristics of the AE-YOLO model under the COCO standard evaluation system, this study systematically analyzed the detection results of seven typical defects based on COCO evaluation indicators. The quantitative analysis data of different types of defects are detailed in Table 3.

Based on the evaluation results in Table 3, the performance of the AE-YOLO model on the PVEL-AD dataset is as follows: mAP value is 89.9%, mAP@0.5 is 89.1%, and mAP@0.75 is 87.5%. The detection performance of medium and large target defects is 56.1% and 34%, respectively, fully verifying the advantage of the Adown module in preserving fine-grained features. The relatively low global average mAP score is mainly attributed to the lack of specific size targets in certain defect categories during the evaluation process. The short circuit lacks medium-sized targets (mAP_m = nan), while the finger and print error both lack large instances (mAP_l = nan). According to the COCO evaluation protocol, these missing dimension annotation samples result in the inability to calculate the corresponding size box indicators, thereby affecting the overall average calculation. This phenomenon reflects the limitations of the dataset rather than fundamental model performance issues.

### 3.4. Visual Analysis

**Figure 12:**
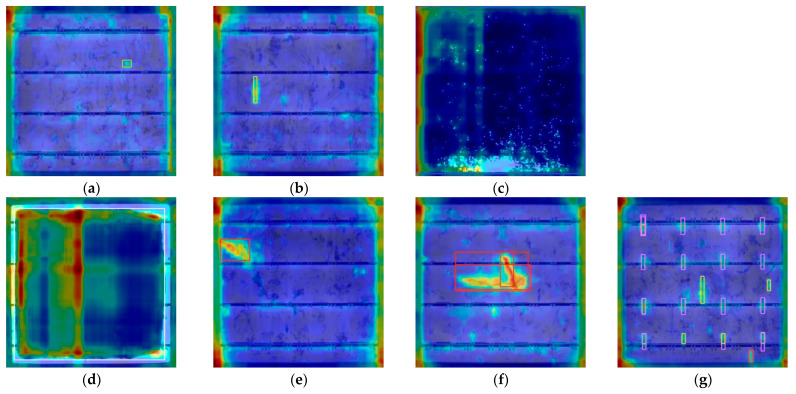
Heat-map effects of various defects before improvement. (**a**) Thick line. (**b**) Finger. (**c**) Short circuit. (**d**) Black core. (**e**) Crack. (**f**) Star crack. (**g**) Printing error.

**Figure 13:**
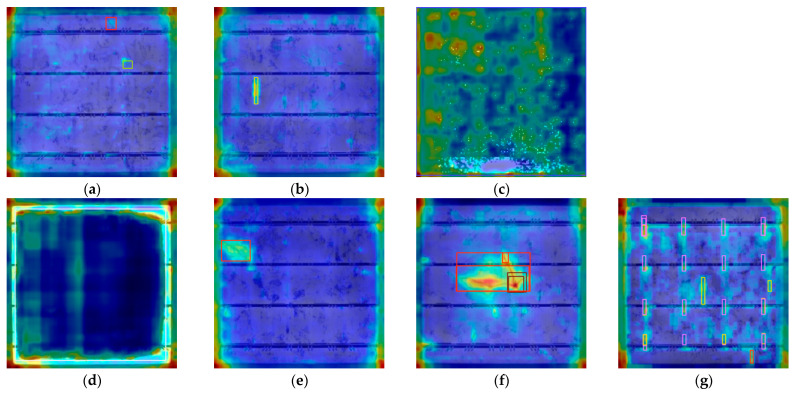
Heat map effects of various defects after improvement. (**a**) Thick line. (**b**) Finger. (**c**) Short circuit. (**d**) Black core. (**e**) Crack. (**f**) Star crack. (**g**) Printing error.

To visually verify the improvement effect of the model, this study used Grad CAM to generate feature heat-map for comparative analysis. The yellow and red regions represent the areas that the model focuses on during classification or prediction, which has a significant impact on the model’s decision-making. The blue and purple areas have a relatively small impact on the decision-making of the model. The comparison of the heat-map of various defects before and after improvement is shown in Figure 12 and Figure 13. Figure 12a–g are the heat-map of various defects generated before improvement, and Figure 13a–g are the heat-map of various defects generated after improvement.

The original YOLOv11 has the problem of attention dispersion. When detecting slender defects such as thick line, the characteristic response shows a discontinuous distribution, indicating insufficient ability to capture key features of the defect. In contrast, the AE-YOLO exhibits significantly optimized attention mechanisms. The improved heat-map shows that the model can generate continuous high response areas along the crack direction for thick line defects, accurately covering the defects. For complex defects such as star crack with multiple branches, each branch area can be activated simultaneously. For categories such as short circuits, the heat-map accurately focuses on the defect subject. This improvement mainly stems from the synergistic effect of the Adown module and the ECA mechanism. The Adown module preserves richer spatial details through dual path feature processing, laying the foundation for precise positioning. The ECA mechanism enhances the response to defect sensitive features through channel calibration, improving the model’s focus on critical areas.

### 3.5. Analysis of Defect Detection Results

**Figure 14:**
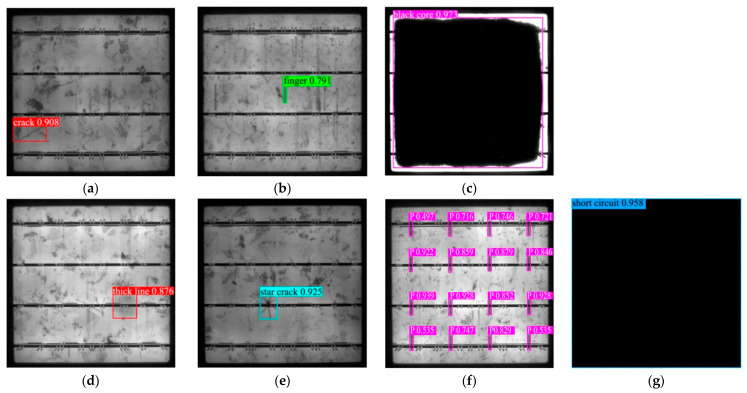
Predicted results. (**a**) Crack. (**b**) Finger. (**c**) Black core. (**d**) Thick line. (**e**) Star crack. (**f**) Printing error. (**g**) Short circuit.

Corresponding detection results are provided for seven types of defects, as shown in Figure 14. It is shown that the model effectively captures slender crack defects with a confidence level of 0.908. The finger defect was also accurately identified with a confidence level of 0.791. The black core defect was accurately detected with a high confidence of 0.973. There are 16 types of printing errors with high confidence level. The model can still provide accurate bounding box localization and reliable classification results when facing complex defects such as star crack, confidence level is 0.925, and the confidence level of the short circuit defects is 0.958.

These visualization results not only demonstrate the localization accuracy and classification confidence of the model in various defect categories, but also highlight its robustness under complex imaging conditions. The AE model can capture the subtle morphological features of slender low contrast crack detection tasks. The results indicate the positive role of the ECA mechanism in enhancing the response of key feature channels, as well as the design advantage of Adown down-sampling module in preserving defect detail structure information. This has jointly promoted the comprehensive performance improvement of the model in multi type defect detection tasks, providing a clear direction for algorithm improvement.

### 3.6. Ablation Experiment

To evaluate the effectiveness of each module in the improved model for photovoltaic panel surface defect detection, the ECA mechanism and the Adown module are successively added to the original YOLOv11 detection model for comparison.

From Table 4, the original YOLOv11 model achieved the mAP@0.5 value of 0.892 on the test set, serving as a baseline reference for subsequent improvement. After introducing the ECA module, the mAP@0.5 increased to 0.895. Although the gain margin is limited, it indicates that cross-channel information interaction can enhance feature representation capabilities. This phenomenon is consistent with the attention mechanism theory proposed by Wang [19], suggesting that local cross-channel interaction helps the model focus on key semantic features. When using the Adown module alone, the mAP@0.5 increased to 0.900, verifying the advantage of the adaptive down-sampling strategy in preserving fine-grained features. Compared to traditional strided convolutions, the Adown dynamically adjusts the down-sampling rate through learnable parameters, effectively mitigating the problem of information loss due to a sudden drop in feature map resolution. When both the ECA and Adown modules are integrated, the model achieves optimal performance, the mAP@0.5 increased to 0.903. Notably, the combined gain exceeds that of a single module, indicating a positive synergistic effect between the two. We speculate that the channel calibration capability of the ECA and the spatial adaptability of the Adown complement each other, enhancing model robustness through multi-level feature optimization.

Experimental results show that: Adown has a greater impact on this task than channel dimension optimization ECA. The module combination strategy can break through the performance bottleneck of a single improvement.

### 3.7. Network Generalization Experiment

To evaluate the generalization ability of the AE-YOLO in different industrial scenarios, we conducted cross domain comparative experiments on the gear surface defect dataset. This experiment strictly maintains the same software and hardware environment configuration and training verification methods as the aforementioned photovoltaic panel defect detection to ensure the comparability of the results. The gear dataset contains three typical defect states: rusted, intact, and damaged.

Meanwhile, according to the comprehensive performance comparison results shown in Table 1, AE-YOLO performs the best in defect detection of photovoltaic panels compared to the baseline model YOLOv11. The aim of this study is to evaluate the generalization ability of the modified model. Therefore, the two representative models mentioned above were selected and validated on the gear dataset. Table 4 is the results of comparison of model on the gear dataset.

As shown in Table 5, AE-YOLO also achieved optimal performance on this new dataset: mAP@0.5 reached 91.20%, an increase of 2.77% compared to the benchmark YOLOv11. Meanwhile, mAP@0.5:0.95 also increased from 67.219% to 71.48%. This result clearly indicates that the efficient feature compression and detail preservation capabilities achieved by the Adown module through dual path design, as well as the channel attention mechanism of the ECA module, are fundamental improvements that do not depend on specific defect types or sensors. They can effectively enhance the feature learning and defect identification capabilities of the model in diverse industrial scenarios, and have good universality and promotional value.

### 3.8. Discussions

This paper proposes the AE-YOLO based on YOLOv11 to solve the problems of multi-scale missed detection in surface defect detection of photovoltaic panels. By introducing the Adown and ECA, it achieves collaborative optimization of model performance and efficiency.

(1) Lightweight and efficiency. The improved model achieves the mAP@0.5 value of 90.3% on the PVEL-AD dataset with 5.3G of computation and 2.1M parameters, showing an 18.9% reduction in parameter quantity and a 17.2% decrease in computation compared to the original YOLOv11, verifying the effectiveness of the Adown module in feature compression and information retention, providing a feasible solution for edge device deployment.

(2) Enhanced multi-scale defect detection capability. The Adown alleviates the problem of fine-grained feature loss caused by traditional down-sampling through a dual-path design that integrates average pooling and maximum pooling. The ECA dynamic ally calibrates key channel features, which improves the detection accuracy of irregular scale defects such as crack.

(3) Module collaborative effect verification. Ablation experiments show that the combination gain of the Adown and ECA exceeded the improvement of a single module. It confirms the complementarity of spatial adaptive down-sampling and channel attention mechanism, which collaboratively optimize the multi-level feature expression.

(4) Industrial application value. Compared with traditional models such as Faster R-CNN, EfficientDet, and SSD, AE-YOLO has significantly improves mAP@0.5 and FPS. By maintaining real-time inference speed, it solves the complex background noise and multi-type defect detection requirements.

(5) Compared with existing research, the AE-YOLO model exhibits certain advantages on the PVEL-AD dataset. As the creator of this dataset, although reference [20] did not provide specific mAP values, it established a benchmark framework for performance comparison. Compared to the traditional feature engineering method Center Pixel Gradient Information to Center-Symmetric Local Binary Pattern (CPICS-LBP) proposed in reference [21], the AE-YOLO end-to-end detection framework demonstrates significant advantages of deep learning. Compared with the most comparable Bidirectional Attention Feature Pyramid Network (BAFPN) [22], the AE-YOLO model mAP@0.5 increase by 2.23%, while significantly reducing the computational load from 370.2G FLOPs to 5.3G FLOPs. This system comparison validates the dual breakthroughs of AE-YOLO in accuracy and efficiency, and the collaborative optimization of the Adown module and the ECA mechanism provides a better solution for defect detection in photovoltaic panels.

The future work will focus on: introducing a dynamic down-sampling rate adjustment strategy to further adapt to defect scale changes. To enhance the joint modeling capability of spatial channel features, a three-dimensional attention mechanism is introduced. To address the data scarcity dilemma faced by small sample defect categories, semi supervised learning methods are combined to break through this bottleneck [23]. At the same time, exploring the adaptability of the proposed algorithm in complex dynamic scenarios can draw on the adaptive signal classification continuous learning method based on selective multi-task collaboration to enhance the flexibility and robustness of the algorithm. Given the current model training’s high dependence on labeled datasets, future research will focus on achieving functional breakthroughs in scenarios with few samples or weak labeling, such as referencing the practical experience of Virtual Signal Large Model (VSLM) [24] in few-sample broadband signal detection and recognition tasks. Furthermore, the issue of label noise cannot be ignored, as it may severely impair the learning performance of the model or even render it ineffective. The next step will focus on researching coping strategies.

## 4. Conclusions

This article proposes an AE-YOLO model based on YOLOv11 for detecting surface defects in photovoltaic panels. By introducing the Adown and ECA, the model improves detection accuracy while maintaining real-time detection performance. The Adown module utilizes innovative dual path design, combined with average pooling preprocessing and branch convolution processing, to preserve the detailed features of defects while implementing feature map down-sampling. This reduces the model parameter count from 2.590M to 2.100M and the computational complexity from 6.4 GFLOPs to 5.3 GFLOPs, significantly improving the computational efficiency of the model. The ECA mechanism enhances the feature response capability to low contrast small defects through a lightweight local cross channel interaction strategy, improving the model’s recognition sensitivity to small defects such as crack. The experimental results on the PVEL-AD dataset show that the mAP@0.5 reaches 90.3%, while maintaining a real-time detection speed of 259.56 FPS. The ablation experiment confirmed the complementarity of the two modules, while cross domain generalization testing further validated the adaptability of the method in different industrial scenarios, particularly on gear defect datasets mAP@0.5 reaching 91.20%. The main contribution of this study is the effective combination of Adown and ECA, which achieves collaborative optimization of detection accuracy and computational efficiency, providing a reliable solution for real-time detection of defects in photovoltaic panels in industrial scenarios. Future work will focus on further optimizing models on edge devices and exploring their potential applications in more complex environments.

## Figures and Tables

**Table 1:** Experimental environment.

Experimental Environment	Version Information
Operating system	Windows 11
CPU	13th Gen Intel Core i7-13700H
GPU	NVIDIA GeForce RTX 4060 Laptop, 8GB Memory
PyTorch	2.1.0
CUDA	12.8
Driver	572.60

**Table 2:** Comparison of different models.

Model	Input	Backbone	Calculation (GFLOPs)	Parameter (M)	mAP@0.5	mAP@0.5:0.95	FPS	mAP@0.5/Watt
Faster R-CNN	600 × 600	ResNet50	370.2G	137.099	0.280	0.225	52.10	0.00403
SSD	300 × 300	VGG16	62.7G	26.285	0.815	0.478	97.20	0.0150
EfficientDet	600 × 600	EfficientNet-D3	5.2G	3.874	0.457	0.306	88.20	0.0226
YOLOv5s	640 × 640	CSPDarknet	16.0G	7.040	0.887	0.60481	172.41	0.0651
YOLOv7-tiny	640 × 640	CSPDarknet	13.2G	6.030	0.859	0.5782	152.67	0.0779
YOLOv11	640 × 640	CSPDarknet	6.4G	2.590	0.892	0.63422	297.98	0.0371
AE-YOLO	640 × 640	ACSPDarknet	5.3G	2.100	0.903	0.63813	259.56	0.0367

Note: 1. This study selected four mainstream object detection algorithms, Faster R-CNN, SSD, EfficientDet, and YOLO, and followed the standard input sizes specified in the original literature of each algorithm in the experiment. Faster R-CNN and EfficientDet are both 600 × 600, SSD is 300 × 300, and YOLO is 640 × 640. 2. Calculation (GFLOPs) is used to measure the computational efficiency of deep learning models, referring to the number of floating-point operations required for the forward inference in units of 10^9^. 3. Parameter (M) refers to the sum of all learnable parameters in the model that need to be optimized through learning algorithms. It is measured in M, which is millions of parameters.

**Table 3:** Verification set AP indicator results (COCO Format).

Category	mAP	mAP@0.5	mAP@0.75	mAP_s	mAP_m	mAP_l
Crack	0.744	0.733	0.699	0.324	0.715	0.823
Finger	0.915	0.904	0.887	0.597	0.936	nan
Black core	0.988	0.987	0.977	nan	0.606	0.988
Thick line	0.882	0.858	0.814	nan	0.897	0.746
Star crack	0.774	0.765	0.758	nan	0.774	0.832
Printing error	1.0	1.0	1.0	nan	1.0	nan
Short circuit	0.99	0.99	0.99	nan	nan	0.99
Global avg	0.899	0.891	0.875	--	0.561	0.34

Note: nan indicates that there are no corresponding size targets (small/medium/large) for this category, and the results are based on the COCO evaluation criteria.

**Table 4:** Results of the ablation experiment.

YOLOv11	ECA	Adown	mAP@0.5
✓			0.892
✓	✓		0.895
✓		✓	0.900
✓	✓	✓	0.903

**Table 5:** Results of comparison of model on the gear dataset.

	Precision	Recall	mAP@0.5	mAP@0.5:0.95
AE-YOLO	0.84499	0.83519	0.91204	0.7148
YOLOv11	0.82777	0.79154	0.8843	0.67219

## Data Availability

Dataset (PVEL-AD): github.com/binyisu/PVEL-AD.
